# A compact and wideband MIMO antenna for high-data-rate biomedical ingestible capsules

**DOI:** 10.1038/s41598-022-18468-2

**Published:** 2022-08-22

**Authors:** Abdullah J. Alazemi, Amjad Iqbal

**Affiliations:** 1 Department of Electrical Engineering, Faculty of Engineering and Petroleum, Kuwait University, Kuwait City, 13060 Kuwait; 2 Centre for Wireless Technology (CWT), Faculty of Engineering, Multimedia University, Cyberjaya, Malaysia

**Keywords:** Electrical and electronic engineering, Biomedical engineering

## Abstract

Due to recent advancements in complementary metal-oxide-semiconductor (CMOS) cameras, transferring high resolution images and videos are possible in wireless capsule endoscopy. High-data-rates communication is required for such data, which is possible using multiple-input-multiple-output (MIMO) antennas. In this paper, a low-sized, compact, high-data-rate, highly isolated two-element MIMO antenna with a large bandwidth has been proposed at 2.45 GHz for wireless capsule endoscopy. The geometry of the antenna ($5\times 4.2 \times 0.12\,\hbox {mm}^{3}$) is kept small using meandered geometry, defected ground structure, and high permittivity of the substrate. A wider bandwidth of 620 MHz (2.15–2.77 GHz) is achieved by exciting dual-modes of the antenna using defected ground structure. Furthermore, a lower mutual coupling between the antennas (30.1 dB at 2.45 GHz) is realized, despite the small edge-to-edge gap of 0.5 mm, using combination of defected ground structure and I-shaped stub. Keeping in mind of system level configuration, this antenna is simulated and measured inside a capsule device by considering effects of the other components and the device itself. The practical measurements are performed by inserting the capsule device (containing the MIMO antenna) inside minced meat. To check the safety and effectiveness of the proposed MIMO antenna, it’s specific absorption rate (SAR) and link budget are calculated and validated. In addition, the $2\times 2$ channel specifications are verified which shows satisfactory performance. This antenna has high channel capacity ($\approx 8.2 \,\hbox {bps/Hz}$ at $\hbox {SNR} = 20 \,\hbox {dB}$) than single-input-single-output (SISO) antennas, thus, is a suitable choice for high-data-rate capsule endoscopic devices. To the best of the authors’ knowledge, this is the first implantable MIMO antenna reported so far with such lower dimension and wider bandwidth.

## Introduction

**Figure 1:**
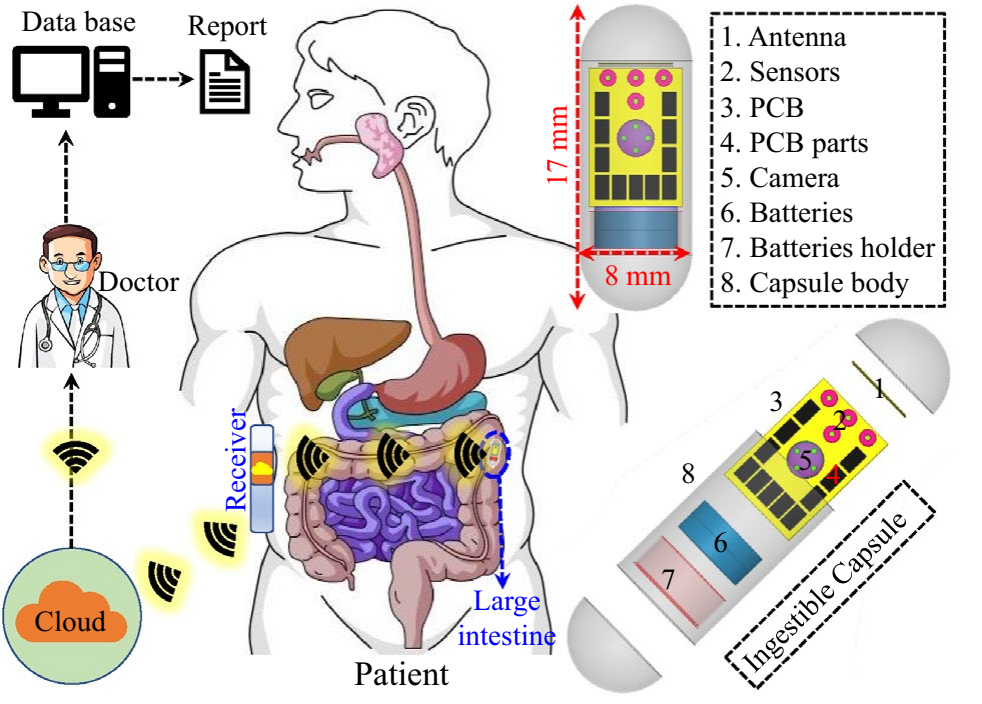
The concept of wireless patient monitoring system using ingestible capsule.

For patients with illnesses related to their internal organs, continuous monitoring of their status requires their physical presence in hospitals for prolonged times. To help such patients and others with similar conditions, recent research efforts targeted the development of wireless patient monitoring systems which collect the required data from the patients in the comfort of their homes without the need to be in the hospital for that purpose. Such systems require a medical sensing device to be inserted inside the body (implantable or ingestible), and this device includes a transmitter to send the data wirelessly to an external device. Next, the received information can be easily transferred to the hospital for real-time analysis and emergency reporting. This concept is demonstrated in Fig. 1 for ingestible capsules as the sensing devices used in monitoring patients with intestinal diseases. A capsule of this type is an integrated system that consists of the required sensors, camera, antenna, batteries and a PCB. The design of such capsules and similar medical sensing devices have been addressed in recent works for different medical applications^1^. For example, monitoring systems for glaucoma intraocular pressure were presented in^1,2^, and for glucose levels in^3,4^.

Besides wireless patient monitoring, implantable medical sensing devices were developed for other medical applications such as recording neural signals and stimulating them^5–7^, heartbeat control^8,9^, and performing capsule endoscopies^10,11^. A key component in the aforementioned implantable systems is the transmitting antenna for which two design parameters are of extreme importance: size and data rate^12^. Size reduction in implantable antennas should be approached using methods that do not degrade the data rate and communication performance. This was achieved in the literature by using the following structures and techniques: reactive loading^13^, substrates with high permittivity^14^, slow-wave structures^15^, and meandered resonators^16^. Another concern while designing implantable antenna is the multi-path distortion (cause by lossy human tissues) inside the human body^17^. One way to alleviate the distortion effects in single-band implantable antennas is to use circularly polarized designs such as those presented in^18,19^. Another way is to deploy multi- or wide-band implantable antennas such as the designs reported in^20,21^ for dual-band operation^22,23^, for triple-band operation^24,25^, for quad-band operation, and^26,27^ for wide-band operation. However, the data rates achieved using the aforementioned techniques are not as desirable for real-time medical applications. To further improve the data rate of implantable antennas, multiple-input multiple-output (MIMO) configuration^28,29^ can be used instead of the single-input single-output (SISO) configuration^30,31^. For example, the MIMO implantable antenna reported in^32^ was used for high-resolution imaging which require high data rates. It used two antennas for transmitting high resolution images captured using wireless capsule endoscope. The main advantage of this antennas was its high data rates compared to other conventional SISO antennas. However, it has demerits of narrow bandwidth, lower isolation and large size. MIMO antennas with two or more elements were also reported in the literature for implantable antenna medical applications^33–35^. In^33^, a dual-element implantable MIMO antenna has been designed using spiral radiator and helix line ground plane. Just like other MIMO antennas, it can support high data rates without extra power and frequency. However, it has the disadvantages of large dimensions, low isolation level and limited 10-dB bandwidth. In^34^, a four-element MIMO antenna has been designed at 2.45 GHz ISM band for implantable medical devices. The antenna is based on electromagnetic bandgap (EBG) structures and a partial ground plane. The main limitations of this antenna is its narrow bandwidth, large dimensions and lower isolation level. In^32^, a dual-antenna system has been introduced for high-data-rate biotelemetry applications. This antenna has advantage in terms of a high isolation level but suffers from a narrow bandwidth and a large size. In^36^, the mutula coupling study of in-body implanted MIMO antenna has been performed. Compare to other antennas, it has relatively small dimensions but at the expense of an extremely narrow bandwidth and large dimensions. The authors of^37^ designed a high-data-rate MIMO antenna for biomedical applications. It has good realized gain but suffers from a narrow bandwidth, a large size and a lower isolation level. In^38^, the authors have designed a MIMO antenna for deep implantable medical devices. It covers a bandwidth of 320 MHz centered at 2.45 GHz. This antenna has several good features (high-data-rate, good channel capacity, and good peak gain etc.), but suffers from large size, limited bandwidth and lower isolation. Although MIMO antennas are proven to increase the data rates, this increase, however, comes at the expense of bulky device^36–38^. Accordingly, using compact element is the main approach to reduce the device size. However, reducing the size of MIMO antennas must be done in a way that maintains high isolation between the elements.

Therefore, the main target of this work is to present a compact, wideband and highly isolated MIMO implantable antenna suitable for medical applications, requiring high data rates. To achieve compactness, the proposed antenna uses a substrate with high permittivity, slots on the ground plane and meandered resonators. Slots and I-shaped stub are introduced in the ground plane of the proposed antenna to effectively isolate the antenna elements. The proposed antenna has a small size ($2.52 \,\hbox {mm}^{3}$) which is one of the smallest in the literature for implantable MIMO antennas. Furthermore, high isolation of 30.1 dB is realized at 2.45 GHz with an edge-to-edge spacing of 0.5 mm between the two antenna elements and both parts are sharing the same ground plane. The proposed antenna operates at 2.45 GHz, posses a wider 10-dB bandwidth of 620 MHz (2.15–2.77 GHz). Furthermore, it maintains a peak realized gain of -20.6 dBi at 2.45 GHz. Health safety parameter, such as specific absorption rate (SAR) analysis is performed to validate patient safety. The result indicates that the 10-g SAR of the antenna is equal to 402.8 W/Kg when a 1 W power is applied. More specifically, the capsule device can handle a maximum input power that is equals to 3.97 mW. This is much higher than the maximum allowable power limit (25 μW). To ensure seamless and high data rate communication, link budget analysis and MIMO channel parameters are investigated. The results show independence between the MIMO elements and seamless high data rates wireless communication.

**Figure 2:**
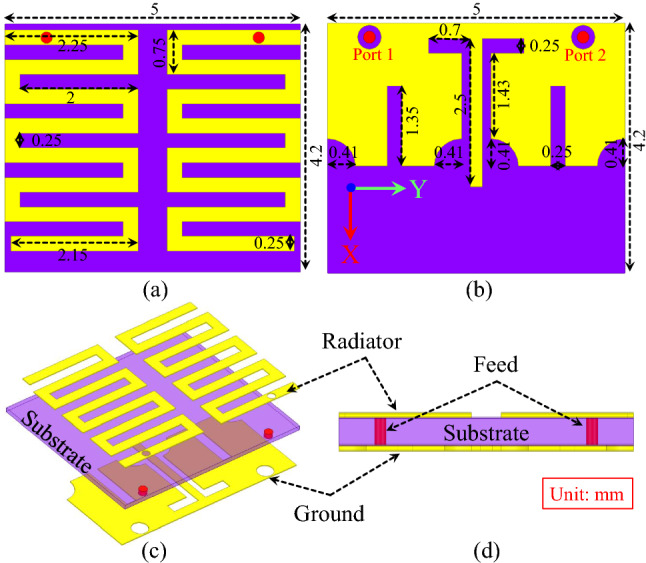
Schematic of the suggested MIMO antenna configuration (**a**) Top view, (**b**) bottom view, (**c**) 3D exploded view, and (**d**) side view.

## Design methodology

### Geometry of the antenna

The layout of the proposed implantable MIMO antenna is shown in Fig. 2 with 3D exploded view (geometry dimensions are shown in millimeters). The antenna is composed of two metal layers with a high dielectric constant substrate in between. The top metal layer consists of two radiating resonators based on meandered transmission lines, while the bottom metal layer is a slotted ground plane that aims at improving the following: (1) improve the antenna impedance matching, (2) increase the isolation between the two MIMO elements, and (3) improve 10-dB impedance bandwidth. Both meandered resonators are fed by a 50 $\Omega$ coaxial cable through SubMiniature version A (SMA) connector attached to the ground. The coaxial cable has an inner and outer conductor diameter of 0.25 mm and 1.5 mm respectively. Furthermore, each cable is 115 mm long. The substrate has a dielectric constant ($\varepsilon _{r}$) of 10.2, loss tangent (tan$\delta$) of 0.0022, and the thickness is equal to 0.12 mm. A High dielectric constant substrate is used in order to achieve smaller size structures to compact the design. The thin substrate layer is envisaged to limit the surface waves leakage between the two elements and improve the isolation. The antenna substrate has dimensions of $5\times 4.2 \times 0.12\,\hbox {mm}^{3} = 2.52 \,\hbox {mm}^{3}$. The compactness advantage of the proposed design is very desirable for capsule implantable devices for deep medical implantable applications.

### Simulation environment

**Figure 3:**
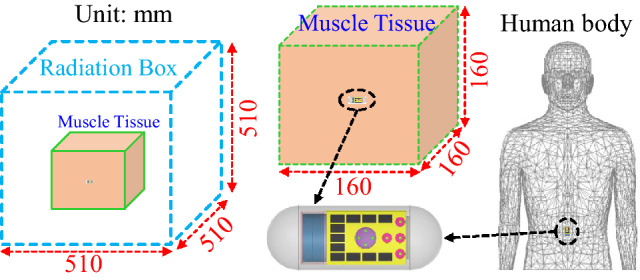
Simulation scenarios of the proposed implantable MIMO antenna system^40^.

The simulation environment is a key stage in this design. This is due to the fact that biomedical implantable antennas are placed inside tissue model or human phantom. In this paper, the implantable antenna is embedded inside a capsule in order to be used in deep-tissue implantation, as illustrated in Fig. 3. In general, deep-tissue capsule devices contain many components such as antennas, CMOS image sensors, surface mounted-lumped elements (capacitors, resistors, etc), Light-emitting diodes (LEDs), batteries, wireless power receivers. All of these components are placed in a thin-walled alumina capsule shell ($\varepsilon _{r} = 9.8$, $\hbox {thickness} = 0.2 \,\hbox {mm}$). The capsule has a cylindrical shape with a diameter of 8 mm and a length of 17 mm.

Figure 3 shows how the antenna is inserted inside a muscle tissue having a dimensions of $160 \times 160 \times 160\, \hbox {mm}^{3}$. The muscle tissue is in cubical shape with a dielectric constant ($\varepsilon _{r} = 52.79$) and conductivity ($\sigma = 1.70 \,\hbox {S/m}$)^39^. In the initial step, the antenna is situated alone inside a cubical muscle in the absence of the capsule implant and placed at a depth of 80 mm. Following which, the suggested configuration is placed with other electronic parts inside the capsule. Then, this configuration is optimized for better results. Finally, abdomen of the human body is chosen as an implantation site where the proposed antenna inside the capsule is placed. It is worthy mentioning that the simulation of both schemes are performed using Ansys high frequency simulation software (HFSS)^40^. In the simulations, the tissue model and human phantom are positioned in a cubical radiation box with the dimensions ($510\times 510 \times 510 \,\hbox {mm}^{3}$), as it can be seen in Fig. 3. The software analysis settings are set to be (a) solution type: terminal mode; (b) solution frequency $= 2.45$ GHz; (c) maximum number of passes $= 20$; (d) maximum Delta S $= 0.01$; (e) Sweeping frequency $= 2$–3.5 GHz with increments of 1 MHz.

### Design evolution stages

**Figure 4:**
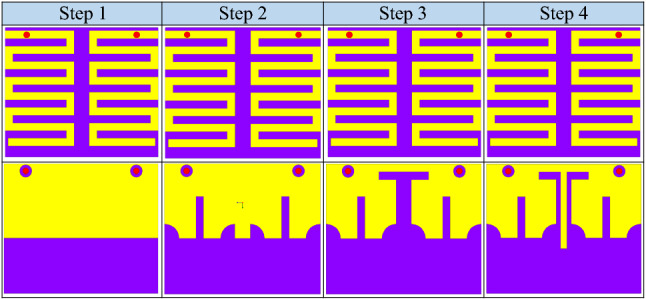
Optimization stages of the suggested MIMO antenna system.

Dissimilar to conventional free space antennas, such antennas operate in heterogeneous body. Consequently, several parameters of the human body affect their performance, thus, obtaining the desired results require several design stages. In order to get the required results, this antenna is designed in four stages, as shown in Fig. 4. During the design evolution stages, several optimization goals are considered. These optimization goals are: (1) compact design, (2) better impedance matching (3) large bandwidth, and (4) high isolation.

#### Step 1 (Initial design)

In the step 1, a conventional half-wave length monopole radiator is designed using Ansys HFSS. The edge-to-edge distance between the monopole radiators is maintained as 0.5 mm. To incorporate extended current path in a small geometry, meandered geometry is selected. The initial dimensions are selected using Eq. (1).

1 $$\begin{aligned} L_{m} = \dfrac{c}{2\times f\sqrt{\varepsilon _{r}}} \end{aligned}$$

where *f* is the resonant frequency of the radiating structure, $\varepsilon _{r}$ is the relative permittivity of the substrate on which radiator is designed, *c* is the speed of light, and $L_{m}$ is the length of the monopole radiator.

**Figure 5:**
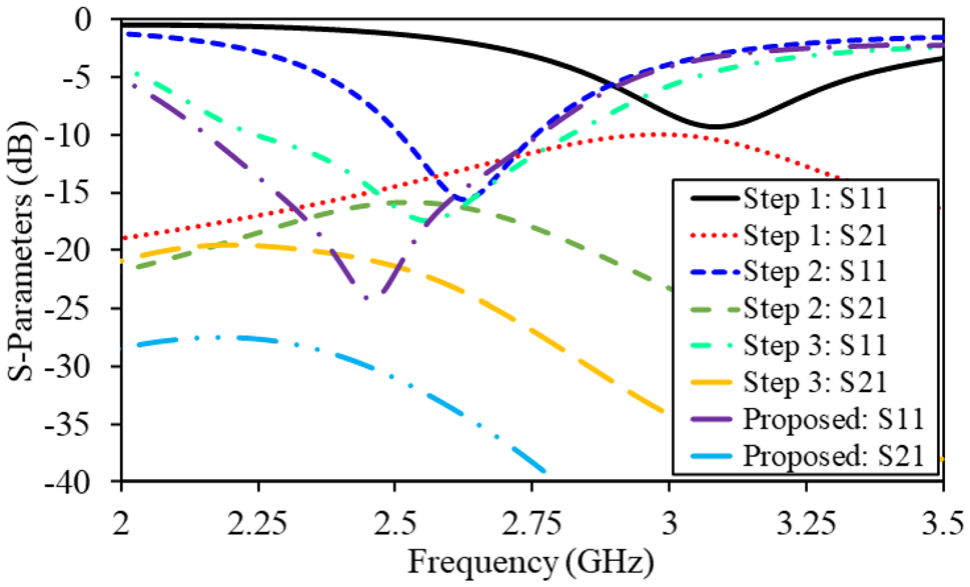
$\hbox {S}_{11}$ and $\hbox {S}_{21}$ of the antenna in optimization stages (optimization goals: compactness, large bandwidth and high isolation).

**Figure 6:**
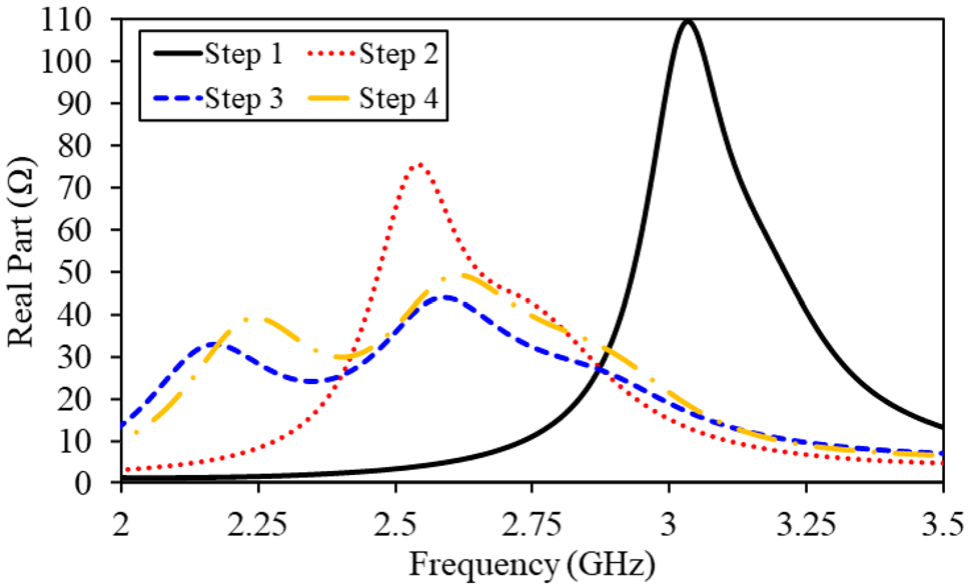
Input impedance (real part) of the antenna in optimization stages.

From Fig. 4, it is obvious that both monopole radiators geometries have shared ground plane. In addition, the S-Parameters of this step are shown in Fig. 5. It can be observed that the antenna resonates at 3.1 GHz and has isolation of 10.3 dB. In this design evolution stage, this antennas suffers from lower impedance matching, large dimensions and higher mutual coupling. Furthermore, impedance of the antenna (real part) is also portrayed in Fig. 6. Evidently, only one mode of the antenna is excited; thus, the antenna has narrow reflection coefficient curve.

#### Step 2 (Slotted ground plane)

In step 2, in order to achieve compact dimensions, several slots in the ground plane are realized, as illustrated in Fig. 4. The realization of additional slots in the ground plane tally extra capacitance^24^. Resultantly, the resonant frequency of the antenna reduces. The impact of additional reactance on the resonant frequency can be elaborated using slow-wave phenomenon. In fact, a conventional antenna can be considered as a series inductor and shunt capacitor. Accordingly, the wave phase velocity can be computed using

2 $$\begin{aligned} v = \dfrac{1}{\sqrt{L_{an}C_{an}}} = \dfrac{c}{\sqrt{\varepsilon _{e}}} = \lambda _{g}f. \end{aligned}$$

Where the propagation velocity is represented with *v*, *c* stands for the speed of light in vacuum, $L_{an}$, $C_{an}$, $\lambda _{g}$ and *f* are inductance, capacitance, guided wavelength, and resonant frequency of the antenna, respectively^15^.

Equation (2) elaborates that reactance of the antenna has inverse relationship with the antenna resonant frequency. In fact, the addition of such slots increase the reactance value; as a results, the antenna is resonating at a lower frequency band and compactness is achieved. The S-Parameters of the second step are demonstrated in Fig. 5. It can be observed that the antenna resonated at 2.6 GHz, with a 10-dB impedance bandwidth of 240 MHz (2.51–2.75 GHz), and isolation of 15.1 dB. With addition of these slots, a miniaturization of 16.12% and isolation improvement of 4.8 dB is achieved. Moreover, the impedance matching of the antenna is also enhanced. In addition, input impedance (real part) of the antenna is shown in Fig. 6 that confirms the miniaturization phenomenon. It can be seen that the fundamental mode of the antenna is shifted to the lower frequencies due to additional slots in the ground plane.

#### Step 3 (Addition of central T-shaped slot in the ground plane)

In the previous design evolution steps (step 1 and step 2), sufficient miniaturization and impedance matching is achieved. However, the antenna still suffer from poor impedance matching, narrow bandwidth and high mutual coupling. In order to increase bandwidth of the antenna and reduce coupling between the radiators, a T-shaped slot is added (Fig. 4). With realization of this slot, second mode of the antenna is excited and bandwidth of the antenna is increased, as shown in Figs. 5 and 6. Similarly, the mutual coupling between the monopole radiators is reduced using this slot. Moreover, further addition of capacitance bring down the resonant frequency to 2.54 GHz. Accordingly, the antenna resonant frequency in step 3 is equal to 2.54 GHz, with a bandwidth of 560 MHz (2.25–2.81 GHz), and an isolation of 21.9 dB.

**Figure 7:**
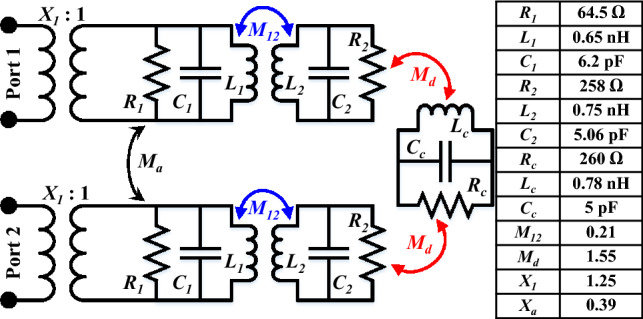
Lumped element model of the proposed wideband implantable MIMO antenna.

#### Step 4 (Addition of central I-shaped stub in the ground plane)

In order to further increase the isolation and impedance bandwidth, a central I-shaped stub is realized in the ground plane. With this addition, the antenna operates at 2.45 GHz, covering an impedance bandwidth of 620 MHz (2.15–2.77 GHz) and isolation of 30.1 dB. In fact, the I-shaped stub enhances the impedance matching, improves 10-dB impedance bandwidth and reduce mutual coupling, as shown in Fig. 5. The combination of T-shaped slot and I-shaped stub activates dual modes of the radiator (as shown in Fig. 6); thus, enhances the 10-dB impedance bandwidth. In addition, they limit the current flow from one radiator to the second; hence, reduce the mutual coupling. By optimizing this antenna through four design evolution stages, all optimization goals (compactness, impedance matching, bandwidth enhancement, mutual coupling reduction) are achieved.

### Equivalent circuit model

In this section, the wider bandwidth and high isolation phenomenon is further elaborated by the aid of lumped element models. As mentioned earlier that this antenna operates at dual excited modes (see Fig. 6); thus, exhibit a wide 10-dB impedance bandwidth. Accordingly, each mode of the antenna is modeled as a parallel RLC circuit, as shown in Fig. 7. Impedance transformers are used for coupling each monopole radiating antenna with the source. Moreover, $M_{12}$ is the inter-mode coupling that is responsible for coupling the two excited modes. In fact, proper coupling between the two modes ($M_{12}$) ensures wide impedance bandwidth. In case of MIMO antennas, there are always some undesirable coupling between the elements. This undesirable coupling between the antenna elements is expressed as $M_{a}$. In order to reduce such coupling, different decoupling techniques are used. In this work, we used defected ground structure and I-shaped stub for coupling reduction. Accordingly, a decoupling RLC circuit ($R_{c}$, $L_{c}$, $C_{c}$) is also added, as shown in Fig. 7. $M_{d}$ refers to the coupling of monopole radiators with decoupling circuit. Finally, this circuit is analyzed and optimized using Advanced Design System (ADS) from Keysight. The optimized circuit values are tabulated in Fig. 7. The S-parameters of the optimized circuit are shown in Fig. 9, which match well with the simulated (EM) and measured results.

## Simulation and experimental results

**Figure 8:**
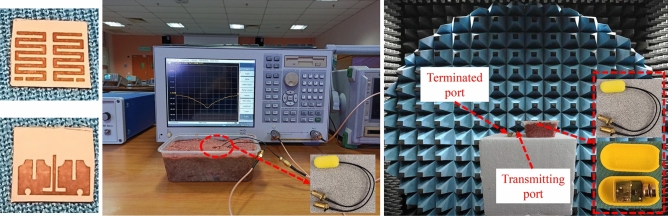
Fabricated antenna and its measurement setup for S-parameters and radiation patterns.

A printed circuit board (PCB) is used to fabricate the wideband MIMO antenna, on a Rogers RO3010 substrate with a high dielectric constant ($\varepsilon _{r} = 10.2$) and low loss tangent (tan$\delta = 0.0022$). The antenna overall size is $5\times 4.2\times 0.12\, \hbox {mm}^{3}$. Ansys high frequency simulation software (HFSS) is used for performing the simulations. Chemical etching process is used in fabricating the antenna metal, stubs, and defected ground structures on a substrate. The capsule shell (with a diameter $= 8$ mm and a length $= 17$  mm) is manufactured with alumina material using 3D printing technology which are taken into consideration during the simulations. Figure 8 demonstrates the measurement environment of the fabricated antenna prototype and the capsule shell. The antenna measured S-parameters are obtained by inserting the capsule into a sample of minced meat. Furthermore, in order to measure the radiation patterns and antenna realized gain, the proposed antenna is placed inside an antenna anechoic chamber.

**Figure 9:**
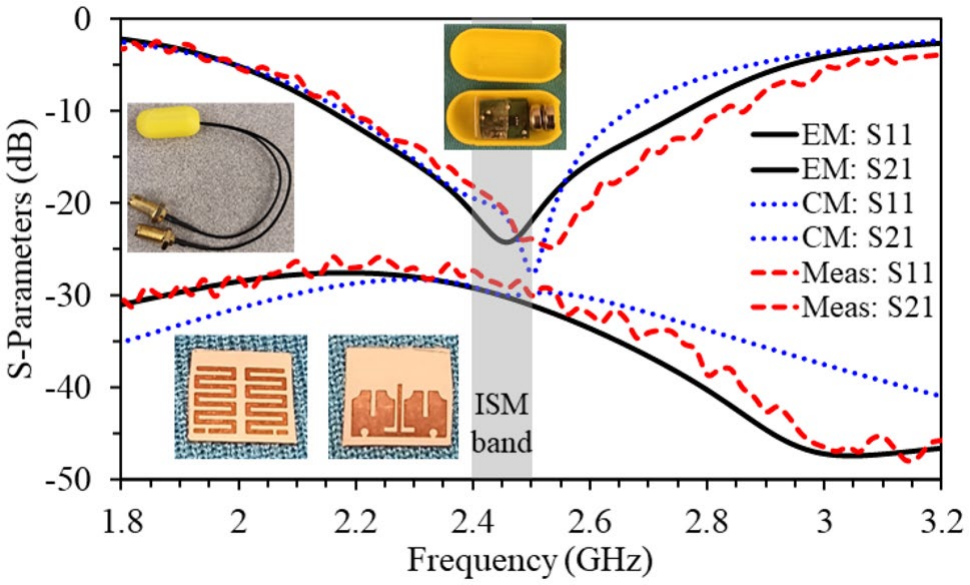
S-parameters (EM: Electromagnetic Model simulations; CM: Circuit model simulations; Meas: Measured).

**Figure 10:**
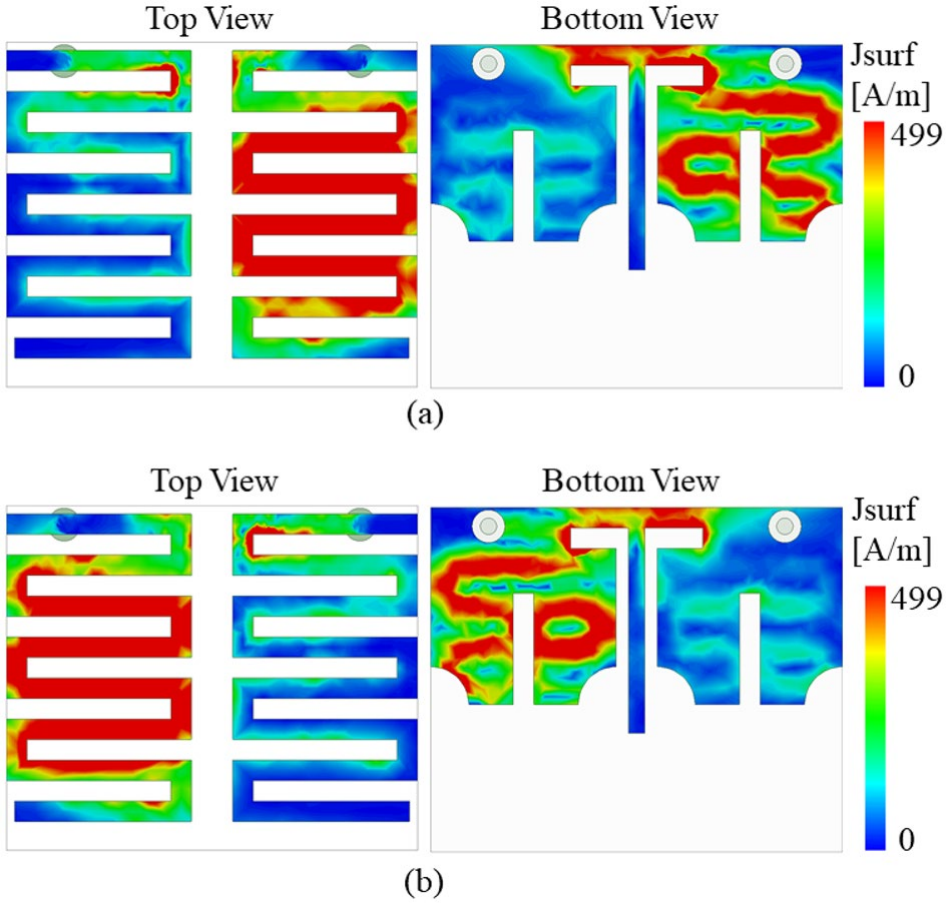
$\hbox {J}_{{\mathrm{surf}}}$ at 2.45 GHz when (**a**) port 1 of the antenna is excited, and (**b**) port 2 of the antenna is excited.

### S-parameters

The proposed wideband implantable antenna’s S-parameters are shown in Fig. 9, which illustrate the results from 3D electromagnetic simulator, the equivalent circuit model (Fig. 7), and the measurements taken from the vector network analyzer. The antenna’s reflection coefficient ($S_{11}$) is matched at the desired center frequency (2.45 GHz) with an achieved 10-dB bandwidth of 620 MHz (2.15–2.77 GHz). The realized mutual coupling ($S_{21}$) between the two antenna parts is lower than 28 dB (with a mutual coupling value of 30.1 dB at 2.45 GHz). Notwithstanding the small edge-to-edge gap of 0.5 mm between the two antenna parts, the mutual coupling between them is very low. The findings show that all the measured S-parameters agree well with the 3D EM simulations and equivalent circuit model simulations.

### Surface current distribution

Figure 10 presents the simulation results of the surface current distribution ($\hbox {J}_{{surf}}$) on both antenna elements. While simulating the design in HFSS, the power is applied to one element while the other element is terminated by a 50 $\Omega$ load. In Fig. 10a, the first element’s port (right element) is excited. The surface current density ($\hbox {J}_{{surf}}$) is observed to be concentrated at the excited patch without a maximum current leakage at the terminated port. The behavior is repeated when the second element’s port is excited (left element), (as shown in Fig. 10b) due to perfect structure symmetry. Therefore, it can be concluded that the isolation between the two antenna parts is performing very well and the current leakage between them is minimal.

### Radiation patterns

**Figure 11:**
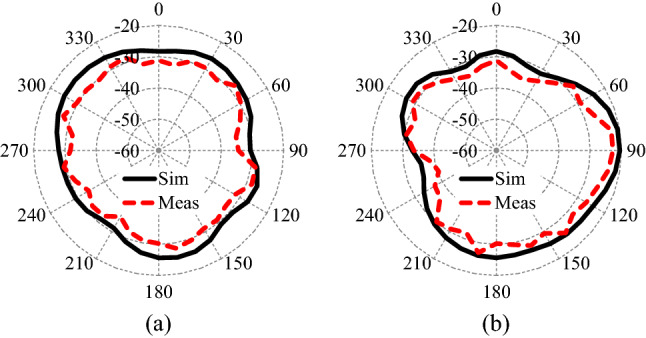
Radiation pattern of the proposed wideband implantable MIMO antenna at 2.45 GHz.

Figure 11 presents the measured and simulated radiation patterns at the antenna’s center frequency (2.45 GHz). The measurement of the antenna radiation patterns are done inside an anechoic chamber at the two main planes (XZ-plane and YZ-plane). The antenna measured radiation patterns are very close to the simulations and have the same behavior at all directions. The radiation patterns are almost isotropic in both principal planes. The isotropic radiation patterns are highly desirable in biomedical implantable devices as they are capable to transmit/receive information waves from all directions. Such radiation patterns are more important for moving implants, such as capsule endoscopes.

**Figure 12:**
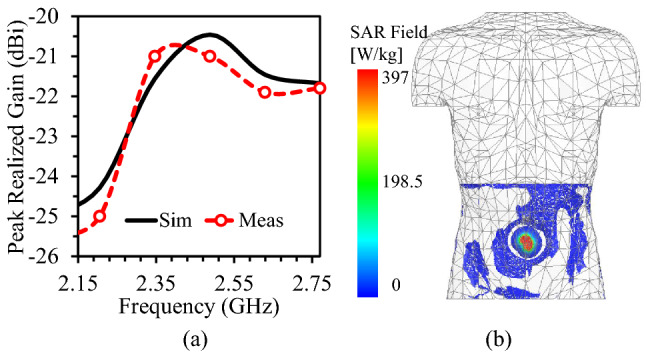
(**a**) Peak realized gain of the antenna as a function of frequency. (**b**) 10-g SAR of the antenna at 2.45 GHz^40^.

Figure 12a illustrates the simulation and measurement results of the proposed antenna’s peak realized gain versus frequency. The results show that the antenna’s peak realized gain is equal to $-20.6 \,\hbox {dBi}$ at 2.45 GHz and the measured gain is almost similar to the simulation ones.

### Specific absorption rate (SAR)

Just like the biocompatibility of antenna and capsule device, it is necessary to ensure human safety in terms of input power and SAR analysis. In fact, the input power and SAR are correlated parameters. More specifically, increasing the input power increases the SAR level. The SAR level can be theoretically calculated using the following equation.

3 $$\begin{aligned} SAR = \frac{\sigma \times E^{2}}{M_{d}} \end{aligned}$$

In the above equation (Eq. 3), $\sigma$ is the conductivity of the tissue, SAR is the specific absorption rate, *E* is the electric field, and $M_{d}$ is the mass density.

As per the IEEE C95.1-2019 standard, the peak SAR value should not exceed 2 W/Kg (for 1W of input power) for the 10-g tissue. During SAR simulation (10-g tissue), each port of the proposed antenna is excited with an input power of 1 W. Moreover, the antenna is placed inside a capsule device and the same device is placed in the abdomen of the realistic human body. With such simulations at 2.45 GHz, it realizes the peak SAR value of 397 W/Kg, as shown in Fig. 12b. Hence, each antenna contributes SAR of 198.5 W/Kg to the total value. Using this SAR value (198.5 W/Kg), each element of this antenna can support 10 mW of input power. It is worthy mentioning that this value is calculated for an input power of 1W; however, only -16 dBm of input power is allowed (ITU-R RS.1346) in real-time applications. The main purpose of keeping such low input power is to reduce interference of the implantable devices with nearby communication devices. Accordingly, the proposed antenna is safe for an input power of 10 mW or less. These results indicate that this antenna is safe to be used in deep-implanted biomedical devices.

**Figure 13:**
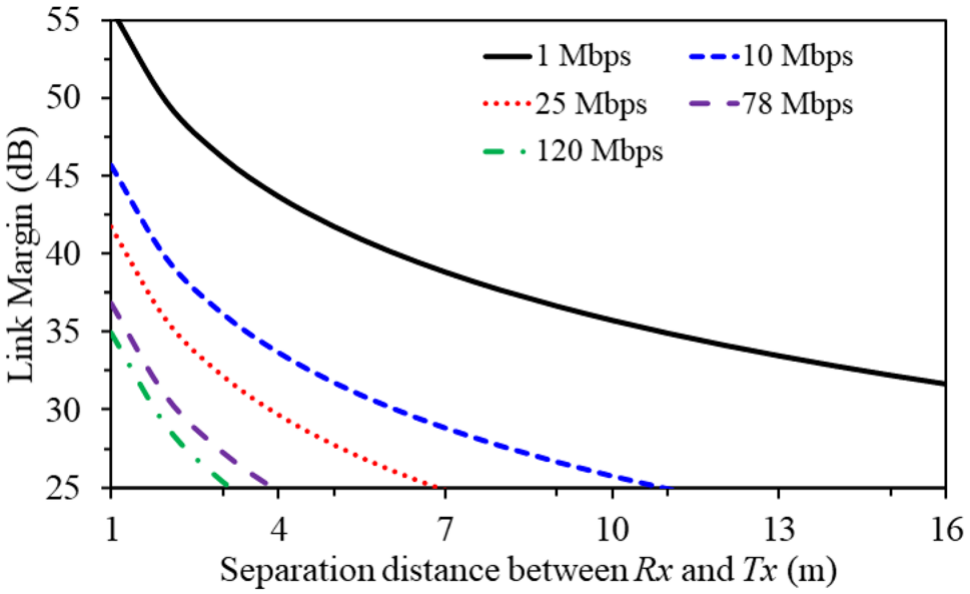
Link margin (in dB) of the proposed wideband implantable MIMO antenna (distance between the transmitter (*Tx*) and receiver (*Rx*) is varied).

### Link budget analysis

An important parameter that has to be accounted for in implantable antennas is the Link budget margin. It covers the aspect of calculating the data transfer rate and coverage area of the antenna prior to implanting the device into a human body. The link budget analysis is performed as per the following: (1) the implantable MIMO antenna is used as a transmitter with a transmit power equals to -16 dBm and a realized gain equals to $-20.6 \,\hbox {dBi}$, (2) a receiver dipole antenna (gain of 2 dBi) is situated at a distance from the transmitter, and (3) the link margin is approximately calculated at different data rates (1 Mbps to 150 Mbps) at 2.45 GHz and calculated at different distances between the transmitter and the receiver using Friis transmission equation^13^. The calculated link margin (in dB) is demonstrated in Fig. 13 versus the distance between the transmitting and receiving antennas (in meters) for various data rate levels at 2.45 GHz. The results show that the output link margin is greater than 25 dB for a 3 m distance between the transmitting and receiving antennas with a high data-rate up to 120 Mbps. The link margin budget is greater than 25 dB for a 7 m with a data-rate level of 25 Mbps. This shows that a satisfactory link margin is achieved to transfer high data rates, thereby ensuring great performance in high loss conditions or any other outer factors.

**Figure 14:**
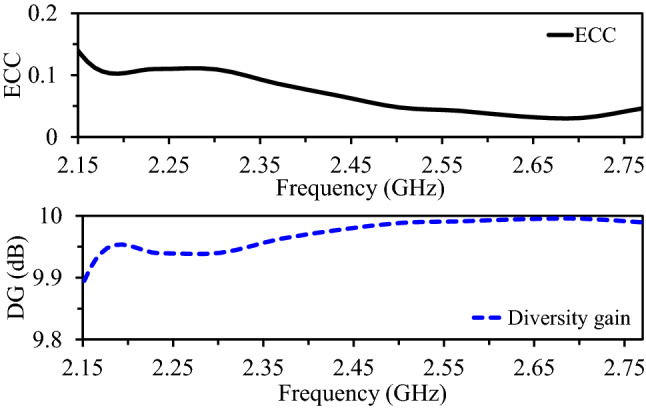
ECC and DG results of the suggested wideband implantable configuration.

### MIMO channel parameters

The MIMO channel parameters are calculated to evaluate the quality of the MIMO system. These parameters include envelope correlation coefficient (ECC), diversity gain (DG), and channel capacity^38^.

The proposed MIMO antenna’s far-fields ECC versus frequency is shown in Fig. 14. The ECC values are calculated from the far-field patterns using the following equation

4 $$\begin{aligned} ECC=\dfrac{|\iint _{4\pi } (\vec {An_{i}}(\theta ,\phi ))\times (\vec {An_{j}}(\theta ,\phi )) \,d\Omega |^2}{\iint _{4\pi } |(\vec {An_{i}}(\theta ,\phi ))|^2\,d\Omega \iint _{4\pi } |(\vec {An_{j}}(\theta ,\phi ))|^2\,d\Omega } \end{aligned}$$

where $\vec {An_{i}}(\theta ,\phi )$ is the far-field 3-dimensional pattern of the first antenna and $\vec {An_{j}}(\theta ,\phi )$ is the far-field 3-dimensional pattern of the second antenna. $\Omega$ is the solid angle. The calculations shows that the ECC values are below 0.11 within the desired wideband frequency range from 2.15 to 2.77 GHz. It is worthy mentioning that a 0.1 ECC value is considered as a fair figure for MIMO systems.

**Figure 15:**
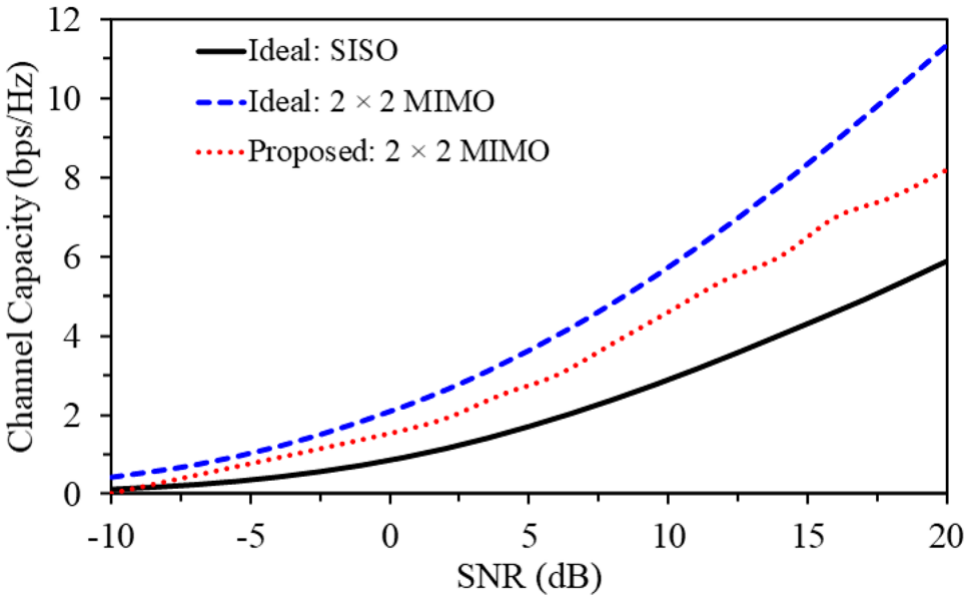
CC versus SNR for the wideband implantable MIMO antenna with three different systems (SISO, ideal MIMO, and proposed MIMO).

The suggested antenna’s diversity gain (DG) versus frequency is shown in Fig. 14. The DG values are calculated from ECC using the following equation

5 $$\begin{aligned} DG=10\sqrt{1-(ECC)^2} \end{aligned}$$

It can be noticed that the diversity gain of proposed antenna is more than 9.9 dB within the desired frequency range (2.15–2.77 GHz), which makes this design a good option for wireless medical applications.

Another important factor to evaluate the proposed MIMO antenna is the channel capacity (CC). The channel capacity of the MIMO topology can be calculated from the antenna radiation patterns through channel matrix which can derived using Eq. (6)^41^.

6 $$\begin{aligned} CC = log_{2}\bigg (det\bigg [I+\dfrac{SNR}{N}HH^{*}\bigg ] \bigg ) \end{aligned}$$

where *CC* is the channel capacity, *SNR* is signal to noise ratio, *N* is noise power, *H* and *I* are channel and identity matrix, respectively.

The channel capacity (in bps/Hz) is plotted (in Fig. 15) versus SNR (in dB) for three different systems, (a) an ideal SISO, (b) ideal $2\times 2$ MIMO antenna and (c) the proposed $2\times 2$ MIMO antenna system. The results show that the *CC* of the proposed MIMO antenna is 8.2 bps/Hz at an SNR of 20 dB, which is greater than the ideal SISO systems (with a *CC* of 5.79 bps/Hz). As a conclusion, the proposed MIMO antenna has better performance compared to the ideal SISO systems, which makes this antenna an excellent candidate for high data rates biomedical applications.

#### Practical implementation

**Figure 16:**
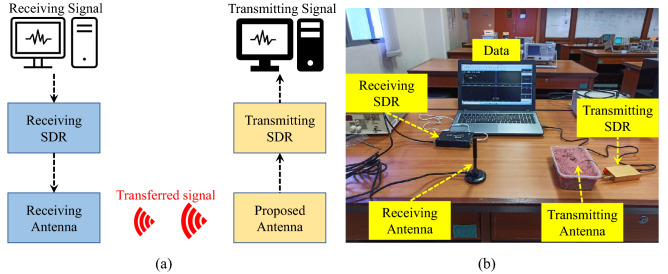
(**a**) Block diagram of experiment. (**b**) Experimental setup for practical measurement of the proposed antenna.

To further understand the working ability of the proposed antenna, experiments are performed using software-defined radios (SDRs), as shown in Fig. 16. In this experiment, two SDRs (one in the transmitting mode and other in the receiving one) are used. Moreover, a dipole antenna, having 2 dBi gain, is used as a receiving antenna. The proposed antenna is placed inside the minced meat. Both SDRs are connected to a laptop, as shown in Fig. 16. A 20 MHz narrow-frequency-modulated (NFW) signal is generated through transmitting SDR and is transmitted through the proposed antenna. A dipole antenna, having 2 dBi gain, is connected to the receiving SDR to receive such signal. The receiving SDR is connected with a laptop. The transmitted signal is successfully received at the receiving end; thus, this antenna has the ability to perform telemetry in real scenarios.

#### Comparison table

At the end, a comparison table (Table 1) is included to summarize the proposed work and compare it with state-of-the-art implantable antennas. Compared to previous published work, our work has advantages in terms of wide impedance bandwidth, compact size and high isolation.

**Table 1:** Comparison of the proposed wideband implantable MIMO antenna with published literature.

References	^17^	^32^	^33^	^34^	^35^	^36^	^37^	^38^	Proposed antenna
Antenna size ($\hbox {mm}^{3}$)	3375	120	307.3	434.6	280	2879	13.03	3.98	2.52
Antenna profile	Non-planar	Planar	Planar	Planar	Planar	Non-planar	Planar	Planar	Planar
Antenna type	Cubical	Spiral	Spiral	EBG	Patch	Conformal	Slotted-Patch	Meandered	Meandered
Tissue	3-Layer	1-Layer	1-Layer	3-Layer	3-Layer	1-Layer	Human Phantom	Human Phantom	Human Phantom
Implant depth	50 mm	100 mm	4 mm	4 mm	3 mm	NR	4 mm & 90 mm	75 mm	80 mm
Elements	4	4	2	4	2	2	2	2	2
Frequency (GHz)	2.45, 5.8	0.432	0.402	2.45	2.4	2.46	0.433	2.45	2.45
Bandwidth (MHz)	88/150	14	14.5	440	210	350	14.67	320	620
Isolation (dB)	$> 32$	12.17	$> 25.6$	$> 15.9$	37	30	26	$>28$	30.1
ECC	$< 0.1$	Not Reported	Not Reported	0.0025	Not Reported	Not Reported	0.1	$<0.1$	$< {{{\textbf {0.11}}}}$

## Conclusion

A low-sized, wideband, compact, and highly isolated two-element MIMO antenna for wireless capsule endoscopy is proposed, designed and measured at 2.45 GHz. Meandered monopole geometry, defected ground structure, and high permittivity of the substrate has been opted to keep the antenna size compact (5 × 4.2 × 0.12 mm^3^). Defected ground structure has been used to excite dual modes in order to achieve wider bandwidth of 620 MHz (2.15–2.77 GHz). Using the combination of defected ground structure and I-shaped stub, a lower mutual coupling between the antennas (30.1 dB at 2.45 GHz) has been realized despite a small edge-to-edge gap of 0.5 mm. Keeping in mind system level configuration, this antenna has been simulated and measured inside a capsule device by considering the effects of other components and the device itself. The practical measurements have been performed by inserting the capsule device (containing the MIMO antenna) inside minced pork meat. It has a measured peak realized gain of $-22.7$ dBi. The ECC, DG, and CC have been verified that show satisfactory performances. The designed antenna has high channel capacity ($\approx 8.2 \,\hbox {bps/Hz}$ at $\hbox {SNR} = 20 \,\hbox {dB}$) than SISO antennas; thus, is suitable option for high-data-rate capsule endoscopic applications.

## Data Availability

All data generated or analysed during this study are included in this published article.
